# Integrated systems biology analysis of acute lymphoblastic leukemia: unveiling molecular signatures and drug repurposing opportunities

**DOI:** 10.1007/s00277-024-05821-w

**Published:** 2024-06-05

**Authors:** Betül Budak, Ezgi Yağmur Tükel, Beste Turanlı, Yağmur Kiraz

**Affiliations:** 1 Department of Bioengineering, Marmara University, Istanbul, Türkiye; 2 Department of Genetics and Bioengineering, Istanbul Bilgi University, Istanbul, Türkiye; 3 Department of Genetics and Bioengineering, Faculty of Engineering, Izmir University of Economics, Balçova, Izmir, Türkiye; 4 Health Biotechnology Joint Research and Application Center of Excellence, Istanbul, Türkiye

**Keywords:** Acute lymphocytic leukemia, Philadelphia-positive acute lymphoblastic leukemia, Biomarkers, Drug repositioning, Systems biology

## Abstract

Acute lymphoblastic leukemia (ALL) is a hematological malignancy characterized by aberrant proliferation and accumulation of lymphoid precursor cells within the bone marrow. The tyrosine kinase inhibitor (TKI), imatinib mesylate, has played a significant role in the treatment of Philadelphia chromosome-positive ALL (Ph + ALL). However, the achievement of durable and sustained therapeutic success remains a challenge due to the development of TKI resistance during the clinical course.

The primary objective of this investigation is to propose a novel and efficacious treatment approach through drug repositioning, targeting ALL and its Ph + subtype by identifying and addressing differentially expressed genes (DEGs). This study involves a comprehensive analysis of transcriptome datasets pertaining to ALL and Ph + ALL in order to identify DEGs associated with the progression of these diseases to identify possible repurposable drugs that target identified hub proteins.

The outcomes of this research have unveiled 698 disease-related DEGs for ALL and 100 for Ph + ALL. Furthermore, a subset of drugs, specifically glipizide for Ph + ALL, and maytansine and isoprenaline for ALL, have been identified as potential candidates for therapeutic intervention. Subsequently, cytotoxicity assessments were performed to confirm the in vitro cytotoxic effects of these selected drugs on both ALL and Ph + ALL cell lines.

In conclusion, this study offers a promising avenue for the management of ALL and Ph + ALL through drug repurposed drugs. Further investigations are necessary to elucidate the mechanisms underlying cell death, and clinical trials are recommended to validate the promising results obtained through drug repositioning strategies.

**Supplementary Information:**

The online version contains supplementary material available at 10.1007/s00277-024-05821-w.

## Introduction

Acute lymphoblastic leukemia (ALL) is a type of malignant hematological cancer characterized by abnormal proliferation and accumulation of lymphoid precursor cells in the bone marrow. Although four-fifths of ALL cases are seen in children, it is known that it is also seen in adult cases and has a more aggressive course. The incidence of ALL follows a dual distribution pattern: the first peak occurs in childhood and the second peak occurs in adults over 50 years of age [1, 2] [1, 3, 4].

ALL is mainly divided into precursor B-cell ALL and precursor T-cell ALL according to antigen receptor rearrangements [5]. B-ALL accounts for approximately 85% of cases, while T-ALL accounts for the remaining 15% [6]. It is known that many different genetic and chromosomal changes play a role in the development of ALL which also contribute to the heterogeneous character of the disease [4, 7]. Chromosomal translocations, which are characteristic features of ALL, can be listed as t(12;21) [*ETV6-RUNX1*], t(1;19) [*TCF3-PBX1*], t(9;22) [*BCR-ABL1*] and MLL rearrangement [8]. The most common of these abnormalities is the formation of the Philadelphia (Ph) chromosome, which occurs as a result of reciprocal translocation. The *ABL1* oncogene located on chromosome 9 and the *BCR* gene located on chromosome 22 come together to form the *BCR/ABL* fusion transcript and the *BCR/ABL* protein that exhibits continuous tyrosine kinase activity. *BCR/ABL* activates mechanisms such as proliferation, growth, metastasis and invasion in the cell through different intracellular signaling pathways [9, 10]. *BCR/ABL*, by its constitutive kinase activity, can also activate the proteins in many different sub-signaling pathways. The pathways that are clinically significant and have been detailed in many different studies are mainly listed as RAS/RAF/MEK/ERK, PI3K/AKT and STAT pathways. Activation of these pathways shows many different cellular effects (the formation of reactive oxygen species, loss of control of the cell cycle, weakening of DNA repair mechanisms, inhibition of apoptosis and autophagy pathways, etc.) and causes the development of leukemias [10–14]. ALLs in this subtype are called Ph + ALL. Although the Ph-chromosome is the most common chromosomal abnormality in ALL, it makes the disease more aggressive compared to Ph-negative cases and is considered a high-risk factor in the clinic. Although the incidence of Ph + ALL increases with age, almost half of the cases are seen in patients over fifty years of age [15].

Looking at the treatment of Ph + ALL; it has been seen that the standard chemotherapy regimen alone is not effective. Understanding the role of Ph + ALL pathophysiology and *BCR/ABL* oncoprotein in leukemogenesis led to the discovery of the drug imatinib mesylate, a first-generation tyrosine kinase inhibitor (TKI). Imatinib functions by binding to the adenosine triphosphate (ATP) binding site of *BCR/ABL* oncoprotein, thereby inhibiting its activity. This inhibition extends to sub-signaling pathways involved in proliferation, division and growth mechanisms [16–18].

Although the use of imatinib in the treatment of Ph + ALL is a breakthrough in the treatment of patients, combining it with different drugs has demonstrated more effective outcomes. The clinical outcome has led to the development of second-generation (nilotinib and dasatinib) and third-generation (ponatinib) TKIs [19–21]. However, the emergence of secondary mutations in *BCR/ABL* leads to TKI resistance, particularly to imatinib [22]. In summary, TKIs have significantly contributed to Ph + ALL treatment, however achieving long-term and permanent success remains challenging due to TKI resistance in the clinical course. Hence, alternative approaches are essential to achieve long-term and persistence success in ALL treatment.

Developing and launching a new drug in cancer treatments requires high costs and time. The risk, cost and time savings provided by drug repositioning bring a new perspective to cancer treatments. Drug repositioning (a.k.a. drug repurposing, reprofiling) is the use of approved or investigational drugs for diseases outside their medical scope. The critical steps in the drug repositioning process are: (a) identification of candidate molecules for use in the targeted disease, (b) testing effectiveness of the drug with in vitro applications and, (c) starting phase 2 clinical trials for the medical indication in which the drug is newly positioned.

Although there are many drug repositioning studies for cancer in the literature, drug repositioning studies for leukemia continue to be added only in recent years. Rapamycin and valporate were the two previously approved drugs for kidney transplant rejection and epilepsy respectively; had been approved later for chronic myeloid leukemia (CML) and acute myeloid leukemia (AML) patients for their potential effect on vital signaling pathways in leukemia progression [23–30]. Perez et al. mentioned that the cyclic AMP (cAMP) pathway may be a significant target for hematological cancers and the importance of examining drugs targeting this pathway with a repositioning approach for the treatment of AML and ALL. Still, this suggestion has not been supported by further studies [31]. Mezzatesta et al. screened repositionable drugs that can be used in the treatment of ALL and showed the effectiveness of anthelmintic drugs on primary ALL cells. In this study, anthelmintic drugs showed anti-proliferative activity even in refractory ALL cells and it was revealed that these drugs were repositionable [32]. Another anthelmintic drug, mebendazole, has been repurposed for use in T-ALL cells and mouse models. As a result, it has been shown that it suppresses cell proliferation through Notch1 and Hes1 inhibition [33]. In another drug repositioning study conducted on T-ALL, mouse and human gene signatures were compared with the healthy T-cell profiles. Genes with altered expression levels were detected in both groups. As a result of various bioinformatic analyses, three FDA-approved drugs were selected and it was determined that all these drugs triggered apoptosis in T-ALL cells [34].

In addition, the activation of alternative signal pathways as the imatinib resistance mechanism in Ph + ALL is mentioned in the literature. In a study, it was shown that signaling of the *RAS/MAPK* pathway, independent of *BCR/ABL*, triggered imatinib resistance in Ph + ALLs through *EphB4* activation, and targeting *EphB4* also broke imatinib resistance [35]. In another study, it was shown that the activation of the *PI3K* pathway, independent of *BCR/ABL*, caused imatinib resistance in a group of resistant Ph + cells, including Ph + ALL cells, and targeting *PI3K* was effective in breaking imatinib resistance [36]. These studies show how targeting imatinib resistance in Ph + ALLs through alternative pathways is a promising approach. In particular, the study of Quentmeler et al. has clearly shown that targeting alternative pathways that have been determined to be involved in resistance, such as *PI3K*, is a correct approach to target *BCR/ABL* independent imatinib resistance [36].

In this study, our primary objective was to propose a novel and effective treatment approach with a drug repositioning strategy for addressing ALL and Ph + ALL. These subtypes of leukemia have notably low long-term survival rates and no new treatment approaches other than existing treatments have been recommended in recent years. Thus, we anticipate a reduction in technical costs, toxic side effects and the time needed to transition to the clinical phase. In addition, this study represents the first application in the literature of drug repositioning for Ph + ALL, a particularly more challenging subtype of ALL.

## Methods

### Gene expression data collection

mRNA gene expression datasets for ALL and Ph + ALL were obtained from Gene Expression Omnibus (GEO) [37]. The keywords “ALL”, “acute lymphocytic leukemia”, “Ph + ALL”, “Philadelphia-positive acute lymphoblastic leukemia”, “pediatric”, “expression profiling by array”, and “expression profiling by high throughput sequencing” were used and datasets with more than one hundred samples were favored for ALL. Six datasets including GSE635, GSE12995, GSE26281, GSE28497, GSE47051, and GSE79533 for ALL, and three datasets including GSE12995, GSE26281, and GSE79533 for Ph + ALL were selected for differentially expressed gene (DEG) analysis. Since control samples were absent in four datasets for ALL and two datasets for Ph + ALL, control samples of the GSE101454 and GSE28497 datasets were selected considering their experimental platform. Details of the selected GEO datasets are listed in Table 1.

**Table 1:** The table of the GEO datasets used in the scope of the study. ALL, Acute Lymphocytic Leukemia; Ph + ALL, Philadelphia-Positive Acute Lymphoblastic Leukemia

Disease	Accession No	Platform	Sample Size		Pubmed ID
			Disease (*n*)	Control (*n*)	
ALL	GSE635	GPL96	173	-	15,295,046 [38]
ALL	GSE12995	GPL96	155	-	19,129,520 [8]
ALL	GSE26281	GPL96	136	-	23,921,123 [39]
ALL	GSE28497	GPL96	284	4	21,487,112 [40]
ALL	GSE47051	GPL570	108	-	24,063,430 [41]
ALL	GSE79533	GPL570	209	3	27,634,205 [42]
ALL	GSE101454	GPL570	-	6	[43]
Ph + ALL	GSE12995	GPL96	20	-	19,129,520 [8]
Ph + ALL	GSE26281	GPL96	18	-	23,921,123 [39]
Ph + ALL	GSE79533	GPL570	17	3	27,634,205 [42]

### Differentially expressed genes at the mRNA level

Each dataset of ALL and Ph + ALL was analyzed independently to identify differentially expressed genes (DEGs) based on the principle of comparing gene expression levels of disease and healthy samples. Datasets without a control group GSE47051 were analyzed with control samples of the GSE101454, while GSE12995, GSE635, and GSE26281 datasets were analyzed with control samples of the GSE28497. Raw data were normalized using Robust Multiarray Average (RMA) [44] and gene expressions were statistically compared with Linear Models for Microarray Data (LIMMA) [45] method under the R/Bioconductor platform (version Rx64 4.2.1) [46] for DEG analysis. Correction of *p*-values applied in multiple hypothesis tests was performed using the False Discovery Rate (FDR) method. Statistical significance was determined in two dimensions by using the thresholds of adjusted *p*-value < 0.05 and 1-fold change (FC). The direction of the DEGs was determined as up-regulated if FC > 1 or down-regulated if FC < 1. Gene nomenclature (Affy ID and gene symbol) was organized using the bioDBnet platform [47]. Since there are multiple datasets analyzed for ALL and Ph + ALL, the mean fold changes of DEGs were statistically calculated using the R/RobustRankAggreg package [48] with an adjusted *p*-value < 0.05 criteria corrected by the FDR method.

### Protein-protein interaction networks

The BioGRID database (v.3.5.184) [49] containing 44,219 protein-protein interactions (PPIs) among 14,373 different proteins was used to identify physical protein-protein interactions associated with ALL and Ph + ALL DEGs. Visualization of PPI networks and calculation of both local and global topological features (degree and betweenness) were applied in Cytoscape software 3.10.0 [50] and CytoHubba plug-in [51]. A dual metric approach was applied to define hub proteins similar to our previous studies [52].

### Reporter molecules associated with DEGs

Reporter regulatory biomolecules associated with ALL and Ph + ALL DEGs were proposed by using the reporter features algorithm [53] adapted in MATLAB 2010. The *p*-values of the reporter molecules were corrected by the FDR method and the *p*-value threshold was considered < 0.05 [54] [55].

### Gene set enrichment analyses

Gene set enrichment analyses (GSEA) were performed to elucidate the functionality of DEGs. The biological processes, molecular pathways, intracellular localizations, and DEG-associated diseases were determined by the Metascape (metascape.org) bioinformatics tool [56]. In the GSEA analyses, the *p*-value threshold less than 0.01 was accepted as statistically significant.

### Identification of the candidate drugs by drug repositioning

The L1000CDS2 [57] comprises a repository of 30,000 drug expression profiles derived from data sourced from the Library of Integrated Network-based Cellular Signatures (LINCS)-L1000 dataset [58]. The primary objective of L1000CDS2 is to facilitate the identification of potential drug candidates for repositioning by leveraging the analysis of up-regulated and down-regulated DEGs within the context of a specific disease. The top 50 drugs ranking by overlap score are collected as repurposed drug candidates.

In addition to drug repositioning based on the modulating gene expression patterns by targeting disease-associated DEGs, we incorporated a network-centric approach called genexpharma [59] tool and utilized hub proteins as drug targets. It encompasses an extensive compendium of 50.304 documented drug-gene interactions and repurposed drug-disease relevance is statistically estimated based on hypergeometric *p*-values. We evaluated ALL and Ph + ALL hub proteins as input for genexpharma query, separately. The threshold was hypergeometric *p*-value < 0.05. Further, all drug candidates manually searched for non-chemotherapeutic nature, and their novelty in the treatment of ALL and Ph + ALL.

This integrated methodology augments the precision and depth of drug repositioning endeavors, thereby enhancing the potential for the discovery of novel therapeutic interventions.

### Cell culture

SUP-B15 (human Ph + B lymphoblast cell line) and Jurkat (human T lymphocyte cell line) cell lines were purchased from ATCC. SUP-B15 cells were cultured in RPMI 1640 (Gibco) supplemented with 20% heat-inactivated fetal bovine serum (FBS, Gibco) and 1% penicillin/streptomycin (Capricorn Scientific) in 5% CO2 at 37 °C. Jurkat cells have the same culture condition as SUP-B15 except for 10% FBS supplementation [60].

### Cell toxicity assays

Thiazolyl blue tetrazolium bromide (MTT) assay was employed to assess the cytotoxic effects of determined drugs on both ALL and Ph + ALL cells whose effectiveness has been demonstrated by bioinformatic analyses. SUP-B15 cells were cultivated in 96 well plates at a concentration of 10,000 cells/well with the administration of Glipizide (0–80 µM). Furthermore, Jurkat cells were cultured in 96 well plates at the same density as SUP-B15 with the treatment of Maytansine (0-2nM) and Isoprenaline (0–50 µM) consecutively. Afterwards, SUP-B15 and Jurkat cells were incubated at 37 °C for 72 and 48 h separately. Thereupon the incubations, MTT was added to each well, and plates were incubated for 4 h. Following that, DMSO was applied to each well, and absorbance measurements at 570 nm were recorded using a microplate reader [61].

## Results

### Determination of DEGs by combining datasets

**Fig. 1:**
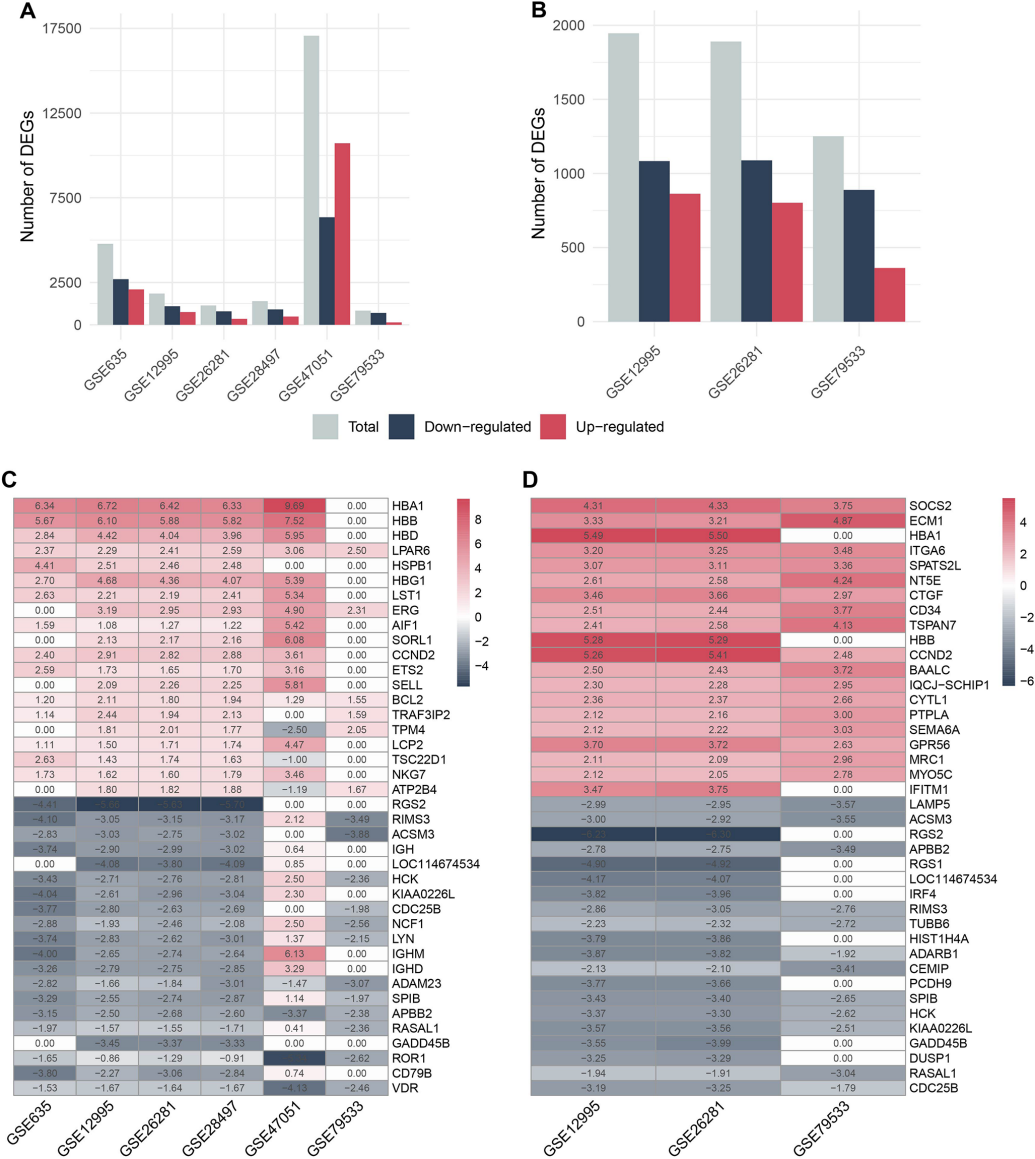
Number of DEGs in the result of DEG analysis of each dataset (**A**) in ALL and (**B**) in Ph + ALL. Red and dark blue bars indicate the number of up-regulated and down-regulated DEGs, respectively. Representation of R/RobustRankAggreg analysis results with heatmap diagram of the top 20 up-regulated and down-regulated DEGs (**C**) in ALL and (**D**) in Ph + ALL. Column names indicate dataset names, while row names indicate gene names. The log2 FC of the gene in each dataset is seen in the boxes. DEG, Differentially Expressed Genes; ALL, Acute Lymphocytic Leukemia; Ph + ALL, Philadelphia-Positive Acute Lymphoblastic Leukemia

In the analysis of gene expression, the LIMMA R package was employed to identify Differentially Expressed Genes (DEGs) in six datasets related to Acute Lymphoblastic Leukemia (ALL) and three datasets related to Philadelphia chromosome-positive ALL (Ph + ALL). The figures presented as Fig. 1A and B illustrate the count of DEGs, up-regulated DEGs, and down-regulated DEGs for both of these diseases.

To enhance the statistical robustness of the DEGs, we utilized R/RobustRankAggreg, a statistical approach that combines data from multiple datasets. This method assigns a ranking to each gene in each dataset and computes the mean FC with a significance threshold of adjp-value < 0.05.

The outcomes of this analysis revealed a total of 698 DEGs in ALL, with 218 genes being up-regulated and 480 genes being down-regulated. For Ph + ALL, there were 100 DEGs, consisting of 67 up-regulated and 33 down-regulated genes. The top 20 up-regulated and down-regulated DEGs, based on their FC, are depicted in Fig. 1C and D.

Notably, the analysis highlights the presence of *AIF1, ETS2, HBG1, LCP2, NKG7, SELL* and *TSC22D1* as up-regulated and *CD79B, IGHM, LYN* and *NCF1* as down-regulated DEGs in the top 20 DEGs for ALL, while these genes do not exhibit differential expression in Ph + ALL. Conversely, *BAALC, CD34, CTGF, CYTL1, IQCJ-SCHIP1, ITGA6, MRC1, MYO5C, NT5E, SEMA6A, SPATS2L* and *TSPAN7* are identified as an up-regulated DEG in Ph + ALL but does not show significant differential expression in ALL.

### Reconstruction of the PPI networks and detection of hub proteins

In the context of our investigation, a total of 6747 Protein-Protein Interactions (PPIs) involving protein-coding DEGs were identified in cases of ALL, while 944 PPIs were discerned in Ph + ALL. Subsequently, we performed an analysis of the top 20 proteins based on both degree and betweenness centrality using the CytoHubba package for both disease categories. Notably, an overlap was observed wherein 15 proteins were found to be common to both ALL and 14 proteins were shared in the case of Ph + ALL, as determined by both degree and betweenness centrality measurements. The set union of proteins identified as top-degree and top-betweenness was designated as the hub proteins for each respective condition.

Consequently, in ALL, 25 hub proteins were ascertained, encompassing ARRB2, BIRC2, BRCA1, CDC20, CHD3, CHD4, EGFR, ERG, HIST1H4A, HSPA4, HSPA5, HSPB1, ITCH, JUN, LYN, MYC, PLK1, PPP1CA, RAD51, RPL37A, SKP1, SOCS2, TP53, UBE3A and YWHAE. In the case of Ph + ALL, 26 hub proteins were identified, including AURKB, BCL2, CCND2, CD44, CDC25B, EFTUD2, ERG, FHL1, FYN, GRB10, HCK, HIST1H4A, HSP90AA1, HSPB1, IRF4, JUNB, MYC, RGS2, SOCS2, TIMM13, TRA2B, TRAF3IP2, TRAF6, TUBB6, VDR and ZMYND11 (Table S1 and S2).

**Fig. 2:**
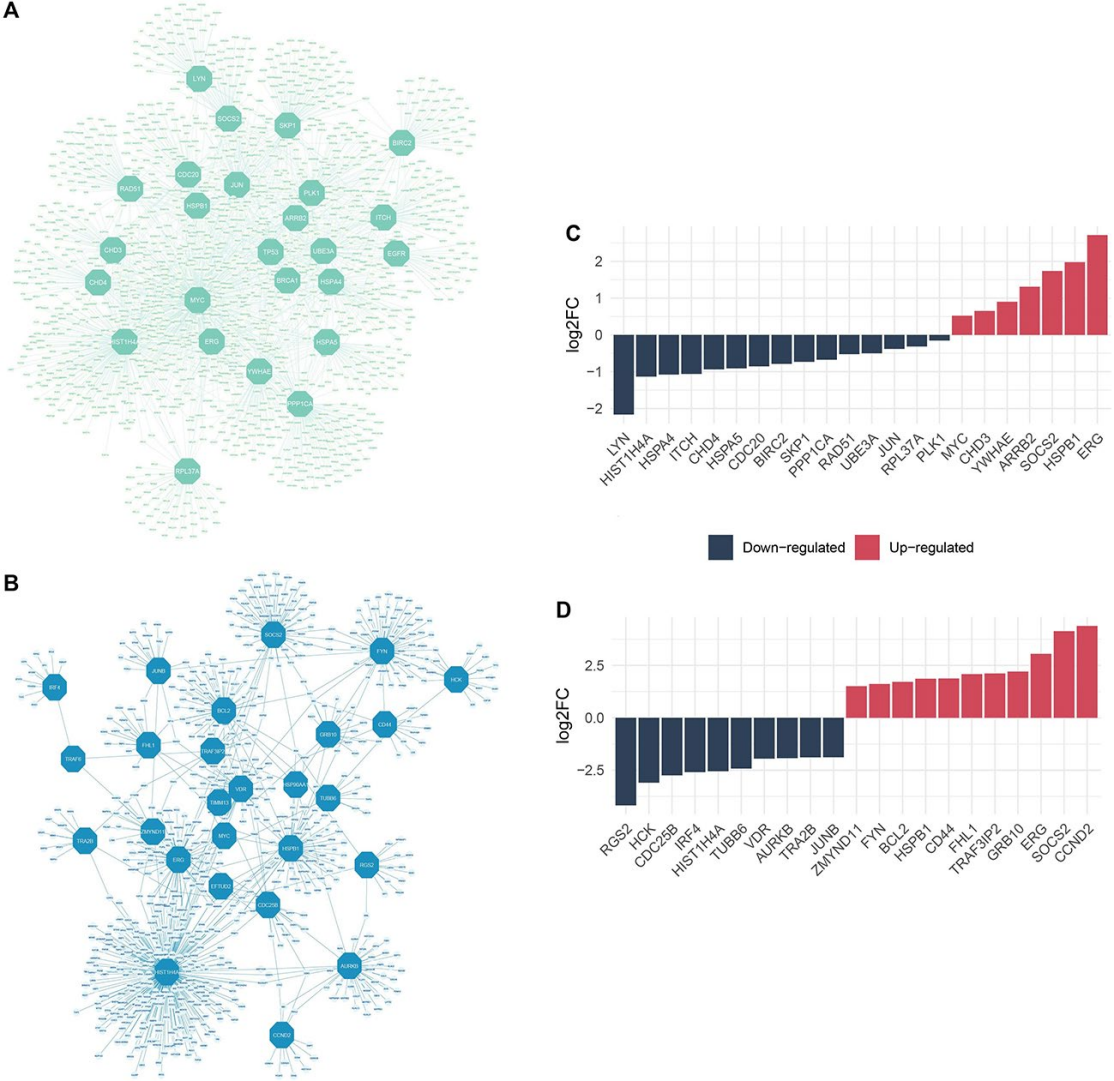
**A**) The interaction network of hub proteins represented by thegreen octagons with other proteins in ALL. **B**) The interaction network of hub proteins represented by theblue octagons with other proteins in Ph + ALL. **C**) Bar plot representation of log2 FC values of DEGs that appear as hub proteins in ALL. **D**) Bar plot representation of log2 FC values of DEGs that appear as hub proteins in Ph + ALL. ALL, Acute Lymphocytic Leukemia; Ph + ALL, Philadelphia-Positive Acute Lymphoblastic Leukemia; DEG, Differentially Expressed Genes

Additionally, 22 of the hub proteins identified in ALL and 21 of those identified in Ph + ALL were also found to be DEGs. To provide a visual representation of the interactions among these hub proteins, we generated a network visualization using Cytoscape, as illustrated in Fig. 2.

### Identification of reporter molecules Associated with DEGs as molecular biomarkers

**Fig. 3:**
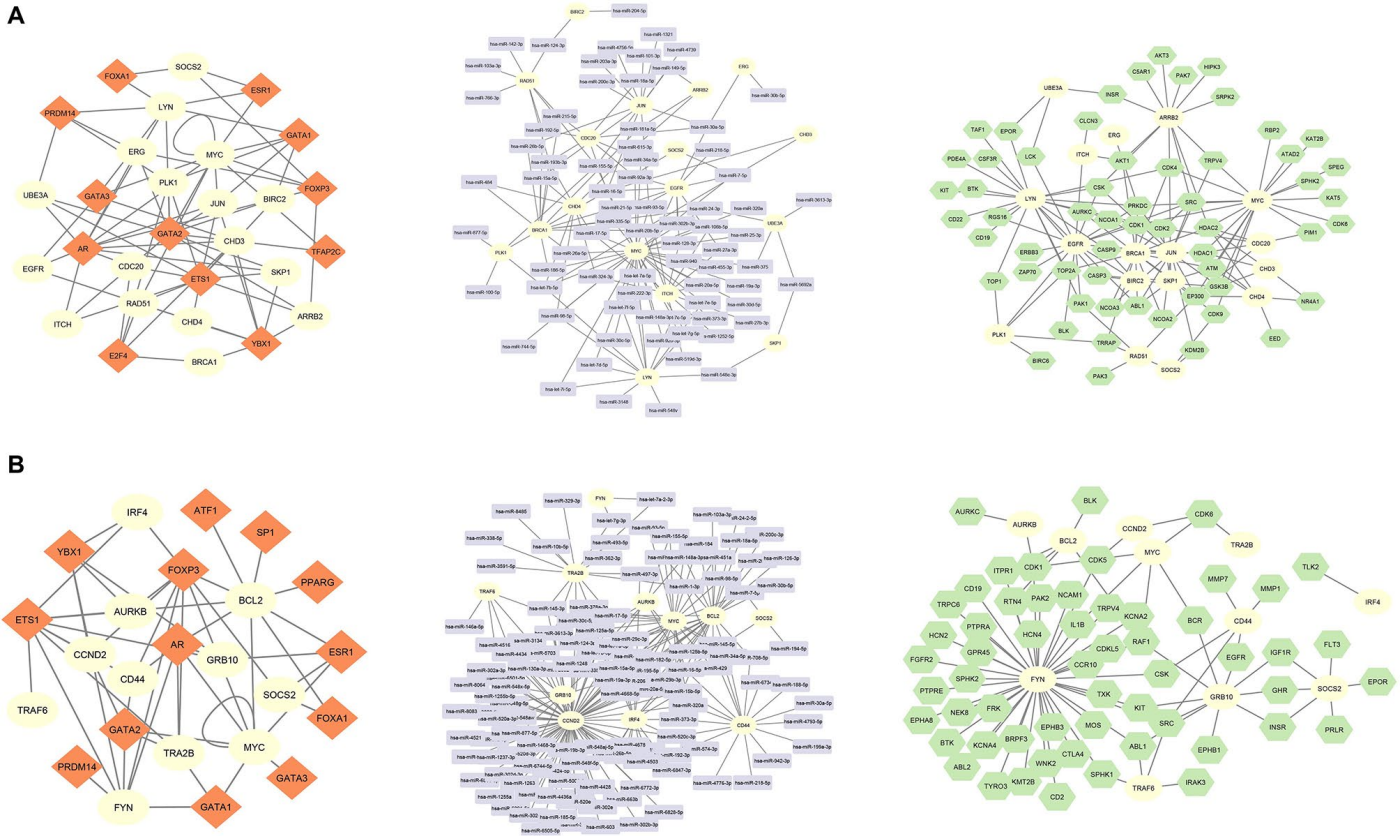
The interaction network of hub proteins with significantly important reporter molecules (**A**) in ALL and (**B**) in Ph + ALL. The yellow circles represent common hub proteins associated with three different reporter molecules. Red diamonds represent transcription factors, lilac quadrilaterals represent miRNAs and green hexagons represent receptors. ALL, Acute Lymphocytic Leukemia; Ph + ALL, Philadelphia-Positive Acute Lymphoblastic Leukemia

Result of the reporter molecules analysis, 14 transcription factors (TFs), 147 miRNAs and 74 receptor molecules were identified as associated with ALL DEGs. These statistically significant molecules interact with ALL hub proteins 96, 213 and 139 times, respectively. The interaction network established by ARRB2, BIRC2, BRCA1, CDC20, CHD3, CHD4, EGFR, ERG, ITCH, JUN, LYN, MYC, PLK1, RAD51, SKP1, SOCS2 and UBE3A hub proteins, which interact with all reporter molecules, was visualized via Cytoscape and presented in Fig. 3A. On the other hand, 21 TFs, 204 miRNAs and 69 receptor molecules were found associated with Ph + ALL DEGs. There are 95, 317 and 101 interactions between these molecules and Ph + ALL hub proteins, respectively. The interaction network of AURKB, BCL2, CCND2, CD44, FYN, GRB10, IRF4, MYC, SOCS2, TRA2B and TRAF6 hub proteins, which interact with all reporter molecules, is depicted in Fig. 3B.

### Gene set enrichment analyses

**Fig. 4:**
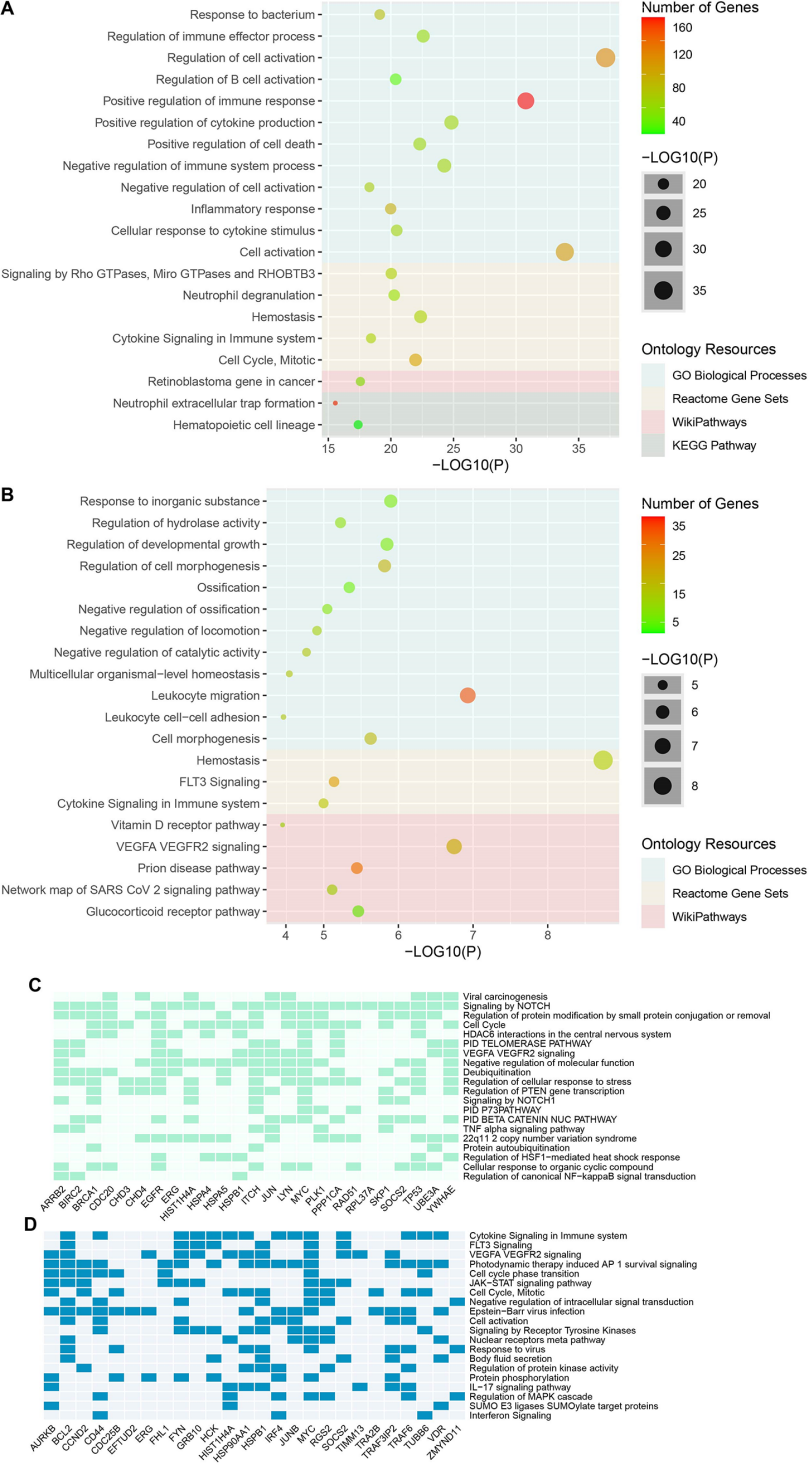
The top 20 biological pathways according to -log10 *p*-value associated with DEGs (**A**) in ALL and (**B**) in Ph + ALL as a result of over-representation analysis. The size of the bubbles represents the *p*-value, while the color scale represents the number of genes involved in the pathways. Heatmap representation of the top 20 pathways in which hub proteins play a major role in the development of (**C**) ALL and (**D**) Ph + ALL. ALL, Acute Lymphocytic Leukemia; Ph + ALL, Philadelphia-Positive Acute Lymphoblastic Leukemia

According to comparative gene set enrichment analysis, cytokine signaling in immune system and VEGFA VEGFR2 signaling pathways were found to be common in both diseases among the 20 important pathways associated with DEGs (Fig. 4A-B). Apart from these, cell cycle, chromatin remodeling, negative regulation of intracellular signal transduction and cellular response to cytokine stimulus were among the significant pathways seen in ALL. Hemostasis, leukocyte migration, response to inorganic substance and regulation of developmental growth were also prominent pathways in Ph + ALL.

On the other hand, Fig. 4C-D show 20 pathways in which ALL and Ph + ALL hub genes play a major role. Again, hub proteins appear to play a common role in the VEGFA VEGFR2 signaling pathway.

### Evaluation of the potential drugs via drug repositioning

In our research, we pursued drug repositioning strategies for ALL and its Ph + subtype through two distinct approaches: targeting DEGs as potential drug targets capable of reversing gene expression patterns and targeting hub proteins as alternative drug targets.

Regarding DEGs, we harnessed the power of L1000CDS2, a drug repositioning tool, to identify 38 unique drugs for regulating the up-regulated and down-regulated DEGs in ALL and 39 unique drugs for Ph + ALL. Remarkably, we uncovered a subset of 7 drugs that were common to both diseases, namely BRD-K68548958, BRD-K80348542, BRD-U86922168, emetine, piplartine, V4877 (verrucarin a) and withaferin-a.

An intriguing observation was made when scrutinizing these drugs: dasatinib and dexamethasone, prominently employed in the primary treatment of Ph + ALL [62, 63], and parthenolide, a natural compound derived from Tanacetum parthenium, renowned for its ability to induce apoptosis in primary human leukemia stem cells [64], were among the identified candidates for Ph + ALL. Homoharringtonine, a plant alkaloid, is an approved drug for the treatment of CML and has been proven to reduce the viability of ALL cell lines when used as a combination therapy [65] [66] also featured in our findings. This convergence with clinically relevant drugs underscores the fidelity of the gene sets employed in our study in faithfully reflecting the underlying pathology.

Turning to the hub proteins, we identified 15 out of 25 hub proteins for ALL, forming a network with 259 interactions involving 186 drugs. For Ph + ALL, 14 out of 26 hub proteins were recognized, constituting a network with 148 interactions that encompassed 132 drugs. Remarkably, in analyzing the drugs shared between both diseases, we identified 65 compounds, including but not limited to dasatinib, fluorouracil, docetaxel, oxaliplatin, sirolimus and paclitaxel, the majority of which belong to the category of antineoplastic agents.

### Assessment of cytotoxic effects of the determined drugs

**Fig. 5:**
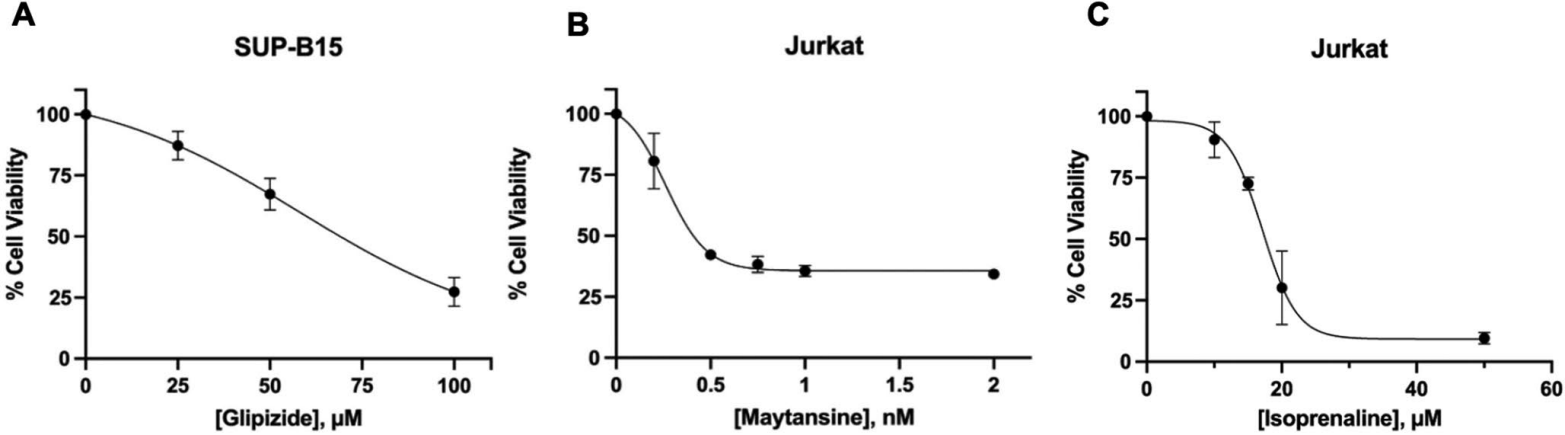
Determination of viability in SUP-B15 and Jurkat cells via MTT assays. (**A**) Effect of increasing doses of Glipizide on SUP-B15 viability. (**B**) Impact of escalated doses of Maytansine on Jurkat viability. (**C**) Impact of escalated doses of Isoprenaline on Jurkat viability. All experiments were performed in triplicate and error bars represent standard deviation (± SD)

Following comprehensive bioinformatic analyses and data interpretations, three drugs were chosen for subsequent in vitro investigations. Specifically, Glipizide were selected for Ph + ALL, represented by the SUPB15 cell line, while Maytansine and Isoprenaline were chosen for ALL, using the Jurkat cell line as the experimental model. To assess the cytotoxic effects of selected drugs on SUPB15 and Jurkat cells MTT assay was utilized. Regarding that, alterations in cell viability induced by three drugs were determined (Fig. 5). Based on the findings from conducted MTT assays, we determined that Glipizide have cytotoxic effects on SUP-B15 cell line, and also Maytansine and Isoprenaline showed similar effects on Jurkat cells. According to the aforementioned assay results, half-maximal inhibitory concentrations (IC50) were established. With reference to that, we examined IC50 values for Glipizide on SUP-B15 cells were 70.42 µM. Likewise, Maytansine and Isoprenaline on Jurkat cells were 439 pM and 13.33 µM. This can be interpreted as Jurkat cells being further sensitive to Maytansine compared to Isoprenaline. Hence, these findings suggest that Glipizide compromised the viability of Ph + ALL cells. Moreover, Maytansin and Isoprenaline induced cell death in the ALL cancer population.

## Discussion

This research performs a comprehensive comparative evaluation of acute lymphoblastic leukaemia (ALL) and Philadelphia chromosome-positive ALL (Ph + ALL) at different molecular levels, including transcriptomics, proteomics and metabolomics. Despite the considerable heterogeneity observed in ALL, its aetiology is associated with a variety of different genetic abnormalities [67]. The most common subtype is Ph + ALL, which is characterized by the *BCR/ABL* translocation and also represents the most aggressive and high-risk variant within the spectrum of ALL subtypes. Ph + ALL poses a major challenge due to the emergence of drug resistance, particularly imatinib resistance, which represents a major obstacle to therapeutic treatment [68].

In response to these challenges, this study attempts to open a new avenue of optimism for the treatment of these diseases through the application of drug repositioning. Notably, to the best of our knowledge, no prior investigations have undertaken a comparative transcriptomic analysis between ALL and Ph + ALL. Furthermore, this study pioneers the utilization of the robust rank aggregation method to identify differentially expressed genes (DEGs) associated with ALL-related diseases.

6 ALL and 3 Ph + ALL datasets were analyzed and differentially expressed transcripts associated with diseases were detected. Statistically significant 698 DEGs for ALL and 100 DEGs for Ph + ALL were found by the robust rank aggregation method. While 32 down-regulated DEGs and 36 up-regulated DEGs were common in both diseases, the down-regulated genes were found to outnumber the up-regulated genes in ALL.

We reconstructed the PPI network of ALL and Ph + ALL. The union of the top 20 proteins according to degree, which refers to the number of interactions of a protein, and betweennes, which refers to the number of connections that a protein establishes with other proteins most shortly, were accepted as hub proteins. We were able to show that these hub proteins play a central role in the development of ALL diseases. Targeting hub proteins with pharmaceutical agents has the potential to halt the effects, progression and spread of the disease. This also makes it possible to target other proteins with which hub proteins interact directly or indirectly and whose expression varies.

ALL is not limited to genetic mutations and changes at the mRNA level. There are also various reporter molecules such as transcription factors, miRNA or receptors that regulate gene expression or are involved in metabolic pathways. For example, the transcription factor MYC, which plays a role in the JAK/STAT signalling pathway, is up-regulated in Ph + ALL and contributes to the survival and proliferation of leukemia cells [69]. Therefore, identifying the relationships between genes with different expressions and reporter molecules sheds light on changes at the transcriptional and post-transcriptional levels in diseases [70]. *ETS1, FOXA1*, *FOXP3*, *MYC, PRDM14* and *GATA* binding protein family stand out as the transcription factors that interact most with the hub proteins we identified in this study. *LYN, MYC, BRCA1, EGFR*, *JUN, ARRB2* are among the most interacting receptor molecules in ALL, while *FYN, GRB10 *are among the most interacting receptor molecules in Ph + ALL. *Let-7* family members, which function as tumor suppressors in different cancers [71] are among the pioneer miRNAs that we associate with ALL diseases. Analyzing the pathways in which genes that cause the development of diseases play a role helps to elucidate the molecular mechanism of diseases. When looking at the statistically significant pathways in ALL, these pathways appear to be cell cycle-related pathwaysand signaling pathways that include signaling by Rho GTPases, Miro GTPases and RHOBTB3 and B cell receptor signaling pathway. Negative regulation and cellular response pathways also play a role in the development of Ph + ALL. Moreover, hub proteins of ALL were mainly involved in signaling by NOTCH, signaling by NOTCH1 and TNF alpha signaling pathway which are pathways that cause ALL cells to survive and grow by escaping the immune system [72, 73]. According to hub proteins of Ph + ALL, the mainly enriched pathways were also IL-17 signaling pathway and JAK-STAT signaling pathway which contribute to development of Ph + subtype and affect the prognosis negatively [74, 75].

The L1000CDS2 and genexpharma tools were used to identify potential drugs for the treatment of ALL and Ph + ALL. When examining the drugs identified as a result of the two analyses, 38 and 186 drugs for ALL, 39 and 132 drugs for Ph + ALL were recommended by L1000CDS2 and genexpharma, respectively. As a result of the analysis, the original indications for testing drugs in vitro were analysed. The information on whether these drugs are used for hematologic cancers or other diseases was considered. It was found that drugs used for solid tumors, hematologic cancers, neurodegenerative and psychiatric diseases come to the fore. Drugs such as methotrexate, an anti-metabolite that inhibits cell proliferation, asparaginase produced from E. coli and mercaptopurine, an anti-leukaemic agent, appear to be among the standard therapies for ALL [76–78]. Dasatinib is an agent approved for single use in the treatment of Ph + ALL and CML patients who are resistant or intolerant to imatinib [79, 80].

In addition, busulfan, bosutinib, nilotinib and imatinib are primary drugs used in many leukemia treatments [81]. These findings prove our study on prognostic markers that we associate with diseases. It has been seen that some of the drugs are currently used cancer drugs (such as bevacizumab, capecitabine, fluorouracil, pertuzumab, sunitinib, trastuzumab), some of them are unapproved drugs under investigation (such as canertinib, geldanamycin, saracatinib) and some of them are examples such as pesticides (Warfarin) whose effects are unknown in humans as drug active ingredients. While determining the drugs to be tested in vitro, importance was given to the selection of drugs that were not used as cancer drugs, drugs that were previously used in the treatment of other diseases in humans but were not tested for ALL or Ph + ALL. As a result, it was decided to test 2 drugs (maytansine and isoprenaline) for ALL and 1 drug (glipizide) for Ph + ALL in vitro. Maytansine is an agent that inhibits cell proliferation by targeting microtubules [66]which targets the EGFR hub protein. Isoprenaline targets the up-regulated hub protein ARRB2 in ALL and is also a β-adrenergic drug used to accelerate heart rhythm [82] [83]. Finally, glipizide targets the GRB10 hub protein which we found to be up-regulated in Ph + ALL compared to healthy samples. It is an anti-diabetic drug of the sulfonylurea class used in the treatment of type 2 diabetes [84].

Subsequently, the MTT assay was performed as a cytotoxicity test to determine the in vitro cytotoxic effects of the preselected drugs. Our results show that all selected drugs, when administered to their respective cell lines at increasing concentrations, resulted in reduced cell proliferation and induced cell death in both ALL and Ph + ALL cell lines (Fig. 5).

This study exhibited certain limitations, primarily stemming from the nature of acute lymphoblastic leukemia (ALL), which is characterized as a hematological disorder rather than a solid tumor. Consequently, the available control samples were notably restricted in number due to the scarcity of suitable comparators for this blood-related ailment. As a result, the sample sizes employed for the analysis of differentially expressed genes (DEG) were considerably smaller for the control group than for the patient group. Additionally, the datasets associated with Philadelphia chromosome-positive (Ph+) ALL, a specific subtype of ALL, were similarly constrained in terms of data volume, thus further constricting the scope of the study.

## Conclusion

The ALL and Ph + ALL transcriptome datasets were analyzed, identifying 698 and 100 disease-related DEGs, respectively. After determining the biological and metabolic processes associated with these genes, the protein interactions and reporter molecules were elucidated. Subsequently, the drugs maytansine, isoprenaline and glipizide were selected for in vitro testing based on optimized parameters. To evaluate the cytotoxic effects of these selected drugs, MTT assays were performed with ALL and Ph + ALL cell lines to assess the cytotoxic effects of these selected drugs and it was determined that all the drugs induced cell death in their respective cell lines. Further analyses to elucidate the mechanism of cell death and clinical trials to validate the exhibited results are recommended for the potential drugs discovered using drug repositioning approaches.

## Electronic supplementary material

Below is the link to the electronic supplementary material.

Supplementary Material 1

Supplementary Material 2

## Data Availability

All datasets used in the study are available at NCBI/GEO database (https://www.ncbi.nlm.nih.gov/geo/). The accession numbers of the datasets are shown in Table 1 in the article.

## Abbreviations

ALL: acute lymphoblastic leukemia
AML: acute myeloid leukemia
ATP: adenosine triphosphate
CML: chronic myelogenous leukemia
DEG: differentially expressed gene
FC: fold change
FDA: Food and Drug Administration
FDR: False Discovery Rate
GEO: Gene Expression Omnibus
GSEA: gene set enrichment analyses
LIMMA: Linear Models for Microarray Data
LINCS: Library of Integrated Network-based Cellular Signatures
MTT: thiazolyl blue tetrazolium bromide
Ph: philadelphia
Ph + ALL: philadelphia chromosome-positive ALL
PPI: protein-protein interaction
RMA: Robust Multiarray Average
TF: transcription factor
TKI: tyrosine kinase inhibitor
