# Correction to: Integrated systems biology analysis of acute lymphoblastic leukemia: unveiling molecular signatures and drug repurposing opportunities

**DOI:** 10.1007/s00277-024-05881-y

**Published:** 2024-07-11

**Authors:** Betül Budak, Ezgi Yağmur Tükel, Beste Turanlı, Yağmur Kiraz

**Affiliations:** 1 Department of Bioengineering, Marmara University, Istanbul, Türkiye; 2 Department of Genetics and Bioengineering, Istanbul Bilgi University, Istanbul, Türkiye; 3 Department of Genetics and Bioengineering, Faculty of Engineering, Izmir University of Economics, Balçova, Izmir Türkiye; 4 Health Biotechnology Joint Research and Application Center of Excellence, Istanbul, Türkiye

**Correction to: Annals of Hematology**

10.1007/s00277-024-05821-w.

In the published paper, Figs. 4 and 5 were mistakenly replaced with Figs. 2 and 3 during proofing stage by the production department of the journal.

We sincerely apologize for the error introduced.

The original article has been corrected.

