# Supplementary material for: Integrated systems biology analysis of acute lymphoblastic leukemia: unveiling molecular signatures and drug repurposing opportunities
Source: Ann Hematol. 2024 Jun 5;103(10):4121–34. doi: 10.1007/s00277-024-05821-w (PMC11512839; doi:10.1007/s00277-024-05821-w)
Supplement: Supplementary file 1 — Supplementary Material 1 [file 277_2024_5821_MOESM1_ESM.docx]

**Supplementary File. Hub Proteins.**

**Table S1:** Top 20 genes in network ALL ranked by degree and betweenness methods.

| **Top 20 in network ALL ranked by Degree method** | | | **Top 20 in network ALL ranked by Betweenness method** | | |
| --- | --- | --- | --- | --- | --- |
| Rank | Name | Score | Rank | Name | Score |
| 1 | HSP90AA1 | 130 | 1 | HSP90AA1 | 808614,3785 |
| 2 | SMARCA4 | 91 | 2 | PCNA | 384131,0274 |
| 3 | PCNA | 89 | 3 | SMARCA4 | 383697,1919 |
| 4 | YWHAE | 68 | 4 | YWHAE | 315450,9685 |
| 5 | CDKN1A | 66 | 5 | CDKN1A | 302312,1022 |
| 6 | YWHAB | 56 | 6 | MYC | 281892,6721 |
| 7 | EZH2 | 55 | 7 | AGR2 | 268114,4671 |
| 8 | HSP90AB1 | 48 | 8 | EGFR | 236435,6407 |
| 9 | PIK3R1 | 47 | 9 | YWHAB | 222448,0111 |
| 10 | CBX5 | 47 | 10 | PIK3R1 | 220526,6802 |
| 11 | JUN | 47 | 11 | EZH2 | 212824,8773 |
| 12 | CDK1 | 40 | 12 | JUN | 187594,2808 |
| 13 | TRAF3 | 39 | 13 | SNCA | 186448,4912 |
| 14 | HSPD1 | 38 | 14 | HSPD1 | 168136,8706 |
| 15 | SNCA | 38 | 15 | CBX5 | 165320,5159 |
| 16 | CDC42 | 37 | 16 | HSP90AB1 | 162431,5041 |
| 17 | CDC5L | 37 | 17 | CDC42 | 159013,9222 |
| 18 | RANBP9 | 34 | 18 | CDK1 | 154256,0769 |
| 19 | RAC1 | 33 | 19 | ZBTB16 | 147549,34 |
| 20 | ZBTB16 | 32 | 20 | TNFAIP3 | 140953,486 |

**Table S2:** Top 20 genes in network Ph+ALL ranked by degree and betweenness methods.

| **Top 20 in network Ph+ ALL ranked by Degree method** | | | **Top 20 in network Ph+ ALL ranked by Betweenness method** | | |
| --- | --- | --- | --- | --- | --- |
| Rank | Name | Score | Rank | Name | Score |
| 1 | RANBP9 | 34 | 1 | RANBP9 | 42532,61967 |
| 2 | LYN | 23 | 2 | LYN | 23908,17913 |
| 3 | CTBP2 | 20 | 3 | MYH10 | 23365,29635 |
| 4 | SYNCRIP | 19 | 4 | ATM | 22230 |
| 5 | INSR | 16 | 5 | CDKN2C | 21994 |
| 6 | IQGAP1 | 13 | 6 | KRAS | 21476,46083 |
| 7 | LGALS3BP | 13 | 7 | CCND2 | 21012 |
| 8 | SYK | 11 | 8 | CDK6 | 20582 |
| 9 | TRAF5 | 11 | 9 | KIAA1429 | 20459,09383 |
| 10 | GNAI1 | 10 | 10 | HDAC6 | 18196,36586 |
| 11 | GRB10 | 10 | 11 | INSR | 16611,45335 |
| 12 | SAP30 | 10 | 12 | ATP2B1 | 16502,29233 |
| 13 | BIRC3 | 10 | 13 | IL6ST | 15113,74858 |
| 14 | SMAD1 | 10 | 14 | SYNCRIP | 14146,75238 |
| 15 | LCK | 10 | 15 | GRB10 | 13087,42507 |
| 16 | RAI14 | 9 | 16 | CDKN2A | 12374 |
| 17 | KRAS | 8 | 17 | CTBP2 | 12330 |
| 18 | LEF1 | 8 | 18 | MYB | 11584,15251 |
| 19 | TRAPPC10 | 8 | 19 | HIPK2 | 11541,25142 |
| 20 | DSP | 8 | 20 | YES1 | 11316,95247 |
